# Associations between psychiatric disorders, COVID-19 testing probability and COVID-19 testing results: findings from a population-based study

**DOI:** 10.1192/bjo.2020.75

**Published:** 2020-07-22

**Authors:** Dennis van der Meer, Justo Pinzón-Espinosa, Bochao D. Lin, Joeri K. Tijdink, Christiaan H. Vinkers, Sinan Guloksuz, Jurjen J. Luykx

**Affiliations:** NORMENT, Division of Mental Health and Addiction, Oslo University Hospital & Institute of Clinical Medicine, University of Oslo, Norway; and School of Mental Health and Neuroscience, Faculty of Health, Medicine and Life Sciences, Maastricht University, the Netherlands; Barcelona Clínic Schizophrenia Unit, Department of Psychiatry and Psychology, Institute of Neuroscience, Hospital Clínic of Barcelona; Department of Medicine, School of Medicine, University of Barcelona; August Pi i Sunyer Biomedical Research Institute (IDIBAPS), Spain; Department of Clinical Psychiatry, School of Medicine, University of Panama, Panama; Department of Psychiatry, Brain Center Rudolf Magnus, University Medical Center Utrecht, Utrecht University; and Department of Translational Neuroscience, Brain Center Rudolf Magnus, University Medical Center Utrecht, Utrecht University, the Netherlands; Department of Medical Humanities, Amsterdam University Medical Center; and Department of Philosophy, Vrije Universiteit, the Netherlands; Department of Psychiatry, Amsterdam University Medical Center; and Department of Anatomy and Neurosciences, Amsterdam University Medical Center, the Netherlands; Department of Psychiatry and Neuropsychology, School for Mental Health and Neuroscience, Maastricht University Medical Center, the Netherlands; and Department of Psychiatry, Yale University School of Medicine, USA; Department of Psychiatry, Brain Center Rudolf Magnus, University Medical Center Utrecht, Utrecht University; Department of Translational Neuroscience, Brain Center Rudolf Magnus, University Medical Center Utrecht, Utrecht University; and Outpatient Second Opinion Clinic, GGNet Mental Health, the Netherlands

**Keywords:** Epidemiology, service users, stigma and discrimination, outcome studies, COVID-19

## Abstract

**Background:**

Many psychiatrists are worried their patients, at increased risk for COVID-19 complications, are precluded from receiving appropriate testing. There is a lack of epidemiological data on the associations between psychiatric disorders and COVID-19 testing rates and testing outcomes.

**Aims:**

To compare COVID-19 testing probability and results among individuals with psychiatric disorders with those without such diagnoses, and to examine the associations between testing probability and results and psychiatric diagnoses.

**Method:**

This is a population-based study to perform association analyses of psychiatric disorder diagnoses with COVID-19 testing probability and such test results, by using two-sided Fisher exact tests and logistic regression. The population were UK Biobank participants who had undergone COVID-19 testing. The main outcomes were COVID-19 testing probability and COVID-19 test results.

**Results:**

Individuals with psychiatric disorders were overrepresented among the 1474 UK Biobank participants with test data: 23% of the COVID-19 test sample had a psychiatric diagnosis compared with 10% in the full cohort (*P* < 0.0001). This overrepresentation persisted for each of the specific psychiatric disorders tested. Furthermore, individuals with a psychiatric disorder (*P* = 0.01), particularly substance use disorder (*P* < 0.005), had negative test results significantly more often than individuals without psychiatric disorders. Sensitivity analyses confirmed our results.

**Conclusions:**

In contrast with our hypotheses, UK Biobank participants with psychiatric disorders have been tested for COVID-19 more frequently than individuals without a psychiatric history. Among those tested, test outcomes were more frequently negative for registry participants with psychiatric disorders than in others, countering arguments that people with psychiatric disorders are particularly prone to contract the virus.

## Background

Coronavirus disease 2019 (COVID-19) caused by the novel SARS-CoV-2 virus strain emerged in Wuhan, China in late 2019 and has since been declared a pandemic.^1^ As of 21 April 2020, there have been over 2.4 million people with COVID-19 and 163 thousand deaths because of COVID-19 worldwide, with about half in the European region. The UK, the fifth most affected country, has reported over 124 000 people with COVID-19 and over 16 500 deaths.^2^ The challenges of this pandemic to health systems, such as the National Health Service in the UK, include workforce scarcity, insufficient infrastructure and limited testing capacity.^3^

During epidemics, people with psychiatric disorders may be more susceptible to infections, experience complications and have more difficulties accessing health services.^4^ Individuals with psychiatric disorders have been shown to have impaired access to somatic healthcare and physical health screening^5,6^ because of a mismatch between patient needs and health systems^7,8^ and stigma;^9^ therefore, many psychiatrists are worried that patients with psychiatric and substance use disorders may be precluded from receiving timely and appropriate testing.^10^

In addition, individuals with a psychiatric disorder may be at increased risk for COVID-19 complications because of comorbid conditions (cardiovascular, respiratory and metabolic conditions, such as obesity)^11–14^ and potential reduced adherence with government measures. However, these issues have remained debatable given the current lack of epidemiological data on associations between psychiatric disorders and COVID-19 testing rates and testing outcomes. More research has therefore been called for to address these questions during and after the COVID-19 pandemic.^15^

## Aims

To address the questions of whether psychiatric disorders have any association with frequencies of testing and the results of such tests, we have targeted a large population-based study (the UK Biobank)^16,17^ to perform association analyses of psychiatric disorder diagnoses with COVID-19 testing probability and such test results. We hypothesised that people with psychiatric disorders are tested for COVID-19 less frequently than people without a psychiatric disorder and that people with psychiatric disorders more frequently test positive than people without psychiatric disorders.

## Method

The full UK Biobank cohort consists of 502 505 individuals recruited between 2006 and 2010, out of which 157 366 participants have information on a mental health questionnaire.^18^ The composition, set-up and data gathering protocols of the UK Biobank have been extensively described elsewhere.^19,20^ We made use of data from UK Biobank participants whose COVID-19 test results were released on 21 April 2020 under application access code 55392.^21^ COVID-19 test results in the UK Biobank, reported in data field 40 100, are mostly derived from samples from nose/throat swabs (or a lower respiratory tract sample in intensive care settings), on which polymerase chain reaction is performed. The UK Biobank data field 40 100 is accompanied by the following statement: ‘We are releasing COVID-19 test results from 16 March 2020 onwards, as after this date UK testing was largely restricted to those with symptoms in hospital. Detection of SARS-CoV-2 from samples taken from hospitalized patients after this date can, at least for now, be viewed as a surrogate for severe disease.’^22^ The first data wave released comprises results from 16 March to 16 April, 2020.^22^ Before analyses, duplicate entries of test results were removed from the COVID-19 testing results by selecting the latest test results for each participant.

UK Biobank has received ethics approval from the National Health Service National Research Ethics Service (ref 11/NW/0382). We used the STROBE cross-sectional reporting guidelines to assess research quality.^23^

For our main analyses we selected ICD-10 diagnoses from UK Biobank data field 41270.^24^ We compared testing frequency and testing outcome in individuals with and without a diagnosis of a psychiatric disorder (F codes). To check whether testing frequency and test results resemble other health conditions, we then included several additional ICD-10 diagnoses in the analyses: (a) individuals with respiratory or cardiovascular diseases as these are particularly at risk for admission to hospital following infection with COVID-19 (ICD-10 codes Jxx and I0x–I7x); (b) people with metabolic diseases as these are highly prevalent among people with psychiatric disorders and may contribute to much of their generally poor health (codes E0x–E1x and E4x–E7x); and (c) those with central nervous system neurological disorders, as a comparison category of diseases resembling psychiatric disorders with regards to symptomatology and hypothesised neurobiological underpinnings (G0x–G4x). A more detailed description on conditions included is available in the supplementary Table 1 (available at https://doi.org/10.1192/bjo.2020.75).

Subsequently, we investigated each of the major psychiatric disorder categories with a prevalence above a threshold of 5% (*n* = 74) among individuals within the subsample that had test results available. We therefore included substance use disorders (F1x), mood disorders (F3x), and anxiety disorders (F40–F41) in the analyses.

All data was analysed in R v3.6.1.^25^ We applied two statistical tests to answer the following primary research questions.

- Are people with a psychiatric disorder more or less likely to undergo COVID-19 testing than people without such a diagnosis? To answer this first question, we used two-sided Fisher's exact tests to examine distributions of individuals tested compared with the full UK Biobank cohort.
- Are people with a psychiatric disorder more or less likely to test positive for COVID-19 compared with those without such a diagnosis? To answer this second question, we ran logistic regression models, using the COVID-19 test results as a dichotomous outcome (negative/positive), with the ICD-10 diagnoses or mental health categories as predictors. We report the change in log odds (β) from these models.

Age, gender, body mass index and assessment centre were used as covariates in the logistic regression models as the first three have been associated with both psychiatric disorders and COVID-19, and assessment centre was added to these models to prevent regional differences having an impact on the results. To assess the robustness of our findings, we further ran a sensitivity analysis additionally covarying for socioeconomic status, as measured through the Townsend Deprivation Index, which has previously been used in the UK Biobank,^26,27^ and pre-existing cardiovascular, respiratory and metabolic conditions as these are commonly observed in people with psychiatric disorders.

Secondary analyses included population-level information on mental health based on mental health questionnaire items asking participants whether they had ever experienced a core symptom of the major mental health categories (data category 136). For example, the two questions on depression were ‘Have you ever had prolonged feelings of sadness or depression?’ and ‘Have you ever had prolonged loss of interest in normal activities?’, tapping into the two core symptoms of major depressive disorder of depressed mood and anhedonia.^28^ If participants had an affirmative response to either of these items, we scored the depression mental health category as present, otherwise as absent. This way, we aimed to examine relationships of the presence versus absence of mental health symptoms (depressive, manic, anxiety, addiction, psychotic experiences, self-harm and happiness items) with both testing probability and testing results in this general population cohort. To test this, we used identical analysis approaches as for the primary analyses, i.e. Fisher's exact tests and logistic regression with the same covariates as mentioned above. We also ran a sensitivity analysis for this secondary analysis by including the abovementioned additional covariates, similar to the primary analyses. We ran these analyses with and without including individuals with a diagnosis of psychiatric disorder to disentangle whether people with any such diagnosis are driving results, and to what extent continuous measures of mental health are associated with COVID-19 testing probability and outcome. Please see the supplementary Tables 2 and 3 for an overview of all mental health items and more information on these analyses.

## Results

The UK Biobank subsample with COVID-19 test results available consisted of 1474 unique individuals. Of these, 842 tested negative (57.1%) and 632 tested positive (42.9%) for the virus. Individuals tested were significantly older than those not tested in the UK Biobank (58.2 years (s.d. = 8.8) *v.* 57.0 years (s.d. = 8.1); *P* = 2.2 × 10^−7^), there were more men among those tested (54.4%) than among those not tested (46.6%), *P* = 2.1 × 10^−9^, and the average Townsend Deprivation Index was lower among those tested than among those not tested (−0.16 (s.d. = 3.53) *v.* −1.30 (s.d. = 3.09), *P* < 10^−16^). There were no significant differences in age (*P* = 0.51), gender (*P* = 0.14) or socioeconomic status (*P* = 0.13) between those testing positive versus negative.

### COVID-19 testing and ICD-10 diagnoses

Individuals with a psychiatric disorder were overrepresented among those tested, making up 23% of this sample compared with 10% in the full UK Biobank cohort (*P* < 0.0001; Table 1). This overrepresentation was similar to, or even higher than, that of people with diagnoses of cardiovascular, respiratory, metabolic, or neurological conditions (Table 1). Furthermore, this overrepresentation was also present for each of the specific psychiatric disorder categories investigated (Table 1).

**Table 1:** Comparison of number of individuals present in the full UK Biobank cohort with those among the COVID-19 tested subset, per diagnostic group, ordered by decreasing ratio^a^

Diagnosis	Individuals in UK Biobank, *n* (%)	Individuals tested, *n* (%)	Ratio tested/in UK Biobank	*P*
Psychiatric disorder	50 506 (10.1)	344 (23.3)	2.32	1.1 × 10^−49^
Neurological	27 950 (5.6)	187 (12.7)	2.28	4.9 × 10^−25^
Metabolic	109 179 (21.7)	580 (39.3)	1.81	8.3 × 10^−53^
Respiratory	88 095 (17.5)	465 (31.5)	1.80	3.8 × 10^−39^
Cardiovascular	178 873 (35.6)	808 (54.8)	1.54	5.1 × 10^−51^
Psychiatric disorder subcategories				
Depression	20 043 (4.0)	156 (10.6)	2.65	1.7 × 10^−27^
Substance use	23 911 (4.8)	173 (11.7)	2.47	9.8 × 10^−27^
Anxiety	11 536 (2.3)	80 (5.4)	2.36	5.5 × 10^−12^

a. The columns indicate the number of individuals with a specific diagnosis in either the full UK Biobank cohort or in the tested subset, and the resulting ratio. The numbers in brackets indicate the corresponding percentage of individuals. The *P*-value is determined by Fisher's exact test.

In Table 1, the numbers of the individual categories add up to more than the total number of tested individuals. This is because of comorbidity i.e. the majority of individuals in the UK Biobank have more than one ICD-10 diagnosis, and particularly individuals with psychiatric disorders have high rates of comorbidity. We have provided an overview of this comorbidity in Supplementary Table 2.

**Fig. 1:**
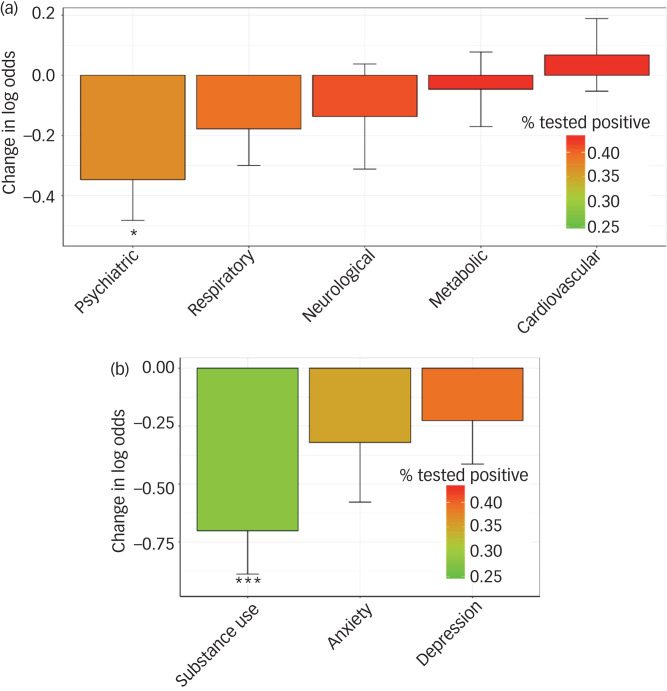
Bar plot of change in log odds for testing positive, by ICD-10 diagnosis.

Among those tested, individuals with a diagnosis of a psychiatric disorder significantly less frequently tested positive for COVID-19 compared with those without such a diagnosis (*P* = 0.01, β = −0.35; Fig. 1a). When looking into specific psychiatric disorders, we found that particularly individuals with substance use disorders were significantly less likely to test positive (*P* = 0.0002; β = −0.70; Fig. 1b). Although people with anxiety and depressive disorders were also less likely to test positive than those without such a diagnosis, these results were non-significant.

(a) The results for the main ICD-10 diagnoses, (b) results for the psychiatric disorders subcategories. Change in log odds is shown on the *y*-axis, with diagnosis on the *x*-axis. Colours indicate per cent that tested positive. *P < 0.05, ***P < 0.0005.

The pattern of results did not change in our sensitivity analysis where we additionally covaried for pre-existing cardiovascular, respiratory and metabolic conditions, as well as socioeconomic status; both general psychiatric disorder and substance use specifically remained significantly associated with test outcome (Supplementary Fig. 1).

### COVID-19 testing and mental health

Please see Table 2 for the prevalence of affirmative responses to mental health items among the full UK Biobank cohort versus those among individuals tested for COVID-19. As shown in the top half of Table 2, there were significant differences in responses between those in the full cohort compared with those in the tested subsample. However, these differences were much smaller than those found for diagnostic categories, and they were no longer significant after excluding individuals with a psychiatric disorder (bottom half).

**Table 2:** Comparison of number of individuals in the full UK Biobank cohort and those among the COVID-19 tested subset, per mental health questionnaire category^a^

Diagnosis	Individuals in UK Biobank, *n* (%)	Individuals tested, *n* (%)	Ratio tested/in UK Biobank	*P*
Happiness	86 010 (54.7)	177 (49.6)	0.91	0.04
Depression	89 034 (56.6)	225 (63)	1.11	0.01
Mania	42 499 (27.0)	109 (30.5)	1.13	0.14
Anxiety	55 199 (35.1)	142 (39.8)	1.13	0.07
Self-harm	30 418 (19.3)	87 (24.4)	1.26	0.02
Addiction	9 382 (6.0)	30 (8.4)	1.41	0.06
Psychotic experiences	7 803 (5.0)	29 (8.1)	1.64	0.01
Excluded individuals with a psychiatric disorder				
Happiness	86 010 (54.7)	149 (49.2)	0.90	0.06
Anxiety	55 199 (35.1)	111 (36.6)	1.04	0.59
Mania	42 499 (27)	87 (28.7)	1.06	0.52
Depression	89 034 (56.6)	185 (61.1)	1.08	0.12
Psychotic experiences	7 803 (5.0)	17 (5.6)	1.13	0.59
Addiction	9 382 (6.0)	22 (7.3)	1.22	0.33
Self-harm	30 418 (19.3)	73 (24.1)	1.25	0.06

a. The columns indicate the number of individuals with an affirmative response to a specific category in either the full UK Biobank cohort, or among the tested subset, and the resulting ratio. The numbers in brackets indicate the corresponding percentage of individuals. The *P*-value is determined by Fisher's exact test. The top half of the table indicates numbers across all participants, the bottom half the numbers after excluding individuals with a psychiatric disorder diagnosis.

**Fig. 2:**
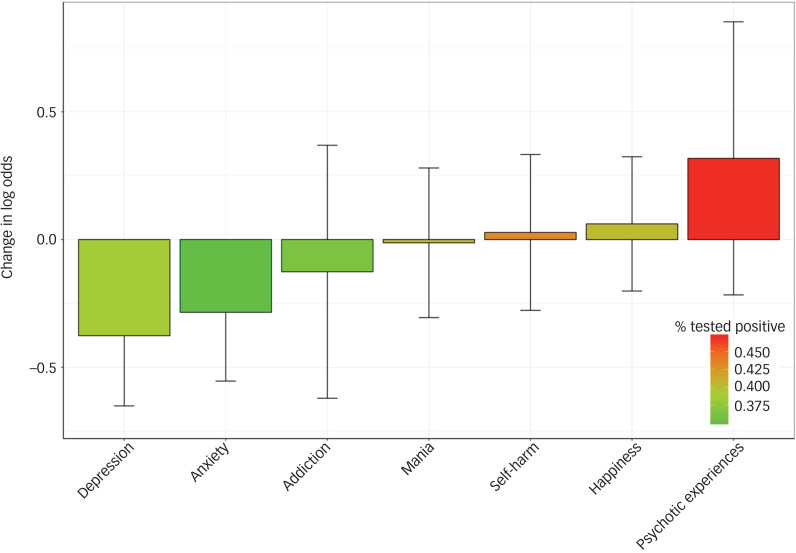
Bar plots of odds ratios for testing positive, per mental health category based on affirmative responses to mental health questions in the mental health questionnaire in the UK Biobank, after excluding individuals with a psychiatric disorder diagnosis.

We further found no statistically significant associations between responses to the mental health items and test results, as shown in Fig. 2. These results stayed the same after correcting for the additional covariates (see supplementary Figures 2 and 3 for the full results).

Odds ratios are shown on the *y*-axis, with the categories on the *x*-axis, colours indicate per cent that tested positive.

## Discussion

### Main findings

Contrary to our hypotheses, we found that individuals with psychiatric disorders have been more frequently tested for COVID-19 compared with those without a diagnosis. Furthermore, among those tested, individuals with psychiatric disorders, substance use disorders in particular, had lower odds of testing positive than individuals without such a diagnosis. We believe these are important findings as they carry the potential to reduce stigma: while people in the general population may be concerned that individuals with psychiatric disorders do not comply with containment measures and are thus susceptible to contract COVID-19 our findings may help counter such concerns. Our findings also may help diminish concerns over limited healthcare access preventing people with psychiatric disorders from undergoing testing.

### Interpretation of our findings

Before we elaborate on explanations for our findings, it is important to contextualise the high rate of positive tests relative to the total test number in the UK Biobank total study population (42.9%). The UK Biobank data provided testing results gathered from 16 March to 16 April 2020, when it was not part of any routine visit or protocol; as per the UK Biobank data-release information provided to researchers, only people showing severe symptoms were tested. Our data-set reflects the beginning of the pandemic in the UK, when testing was largely restricted to those with symptoms in hospital, i.e. people with severe cases, and when testing capacity was low. In this regard, the UK Biobank states that SARS-CoV-2 testing can be viewed as a surrogate for presentation of severe COVID-19 symptoms.^22^

Possibly, people with psychiatric disorders are being tested more frequently because of comorbid conditions, higher levels of anxiety about contracting COVID-19 or perhaps a combination of both. Furthermore, referring physicians’ concerns about COVID-19 in people with psychiatric disorders may contribute to relatively high testing rates. Such reasoning may also, at least in part, explain higher rates of negative COVID-19 test results in people with psychiatric disorders.

Frequently observed negative test results could also be related to their higher chances of somatic illness relative to individuals without such a diagnosis or even with other pre-existing conditions. For example, patients with psychiatric disorders may present to hospitals with COVID-19-like symptoms but could be presenting an exacerbation of chronic pulmonary disease related to high rates of smoking, metabolic syndrome and sedentary lifestyle, further aggravated by late presentation to healthcare services because of poor living conditions or lack of social support. Such situations in patients with a history of psychiatric illness may lead to higher likelihoods of presentations of acute somatic illness requiring admission to hospital and thus, from March 2020 onwards, COVID-19 testing.

A final underlying reason for the low chances of positive COVID-19 testing in patients with a psychiatric history may be that they live relatively more socially isolated than people without such diagnoses. A non-controlled study has shown people with psychosis and mood disorders have about 1.7 social contacts in a week outside home, workplace or healthcare settings, and moderate feelings of loneliness.^29^ Furthermore, a study on the relationship between living alone and psychiatric disorders using the 1993, 2000, and 2007 National Psychiatric Morbidity Surveys in the UK found a positive association between both variables that was up to 84% explained by loneliness.^30^ Among young adults in modern Britain, relatively lonely individuals have been shown to be more likely to have depressive, anxiety, and alcohol use disorders.^31^ Thus, lonelier, more socially isolated people such as those with psychiatric disorders may normally, and now even more so, be in confinement and have less social contact than people without psychiatric disorders, reducing the likelihood of testing positive for COVID-19.

Our results also show the frequency of individuals with a neurological condition undergoing testing being high, comparable with psychiatric disorders in the UK Biobank sample. This could be explained from a symptom-level perspective: psychiatric disorders show the most substantial overlap with neurological disorders regarding symptom domains such as cognitive function, behavioral alterations and mood. One example would be the high rates of depressive symptoms in Parkinson's disease^32^ and multiple sclerosis.^33^ Furthermore, dementias and other types of brain disorders causing behavioral symptoms are grouped within psychiatric disorders in ICD-10;^24^ these show high degrees of symptom overlap with neurodegenerative disorders, especially regarding mood and cognitive functioning.^34,35^ Therefore, people with neurological conditions, either comorbid with psychiatric conditions or presenting symptomatology overlapping with psychiatric symptoms, being both within the realm of brain functioning, would be expected to be affected similarly in the context of COVID-19.

Furthermore, when looking at the symptom level in the UK Biobank sample (mental health questionnaire items), no relationship was found between testing likelihood or outcome and continuous measures of depressive, manic, anxiety, addiction, psychotic experiences, self-harm or happiness items in those without a past or current diagnosis of psychiatric disorder. Therefore, people with clinical cases of psychiatric disorders, and not subsyndromic individuals, appear to drive our primary findings. Statistical power may currently hamper these analyses.

### Limitations

Limitations of this study include the relatively small sample size, the fact that the UK Biobank is not fully representative of the general population,^36–38^ absence of replication in other cohorts and lack of information on indications for testing at the level of the individual. The small sample size precluded individuals with diagnoses of some less prevalent psychiatric disorders, such as schizophrenia, schizoaffective disorder and bipolar disorders, to be represented in the analyses. Furthermore, assessment centre was used as a proxy for geographical location, and this variable was set at the start of recruitment, for example if individuals moved after the initial assessment this was not possible to take this into consideration.

Finally, detailed clinical information on indications for testing are unavailable for each individual, precluding us from running subgroup analyses per clinical indication. Nonetheless, the UK Biobank data gathered testing results from 16 March 2020 onwards when it was not part of any routine visit or protocol; as per the UK Biobank data-release information provided to researchers, only people showing severe symptoms were tested. This makes it relatively likely that patients were not tested routinely prior to admission for psychiatric reasons. Furthermore, although we believe having testing rates of other complementary exams would have been helpful to compare the COVID-19 testing with routine testing, we do not have information on other diagnostic procedures during admission, such as urine toxicology.

### Implications

Despite the aforementioned limitations, two preliminary conclusions can be drawn based on the current data-set given the convergence of findings for a range of psychiatric disorders and similarities between testing probabilities. First, individuals with a psychiatric disorder are not less likely to undergo testing for COVID-19 than those without psychiatric disorders. Second, patients with psychiatric disorders do not test positive more frequently than people undergoing testing without such conditions. We encourage other researchers to perform similar analyses in other cohorts, as well as further research when more data from the UK Biobank become available, for example into associations between extended psychiatric symptom-level data, COVID-19 symptom severity and mortality.

## Data Availability

UK Biobank data are available through a procedure described at http://www.ukbiobank.ac.uk/using-the-resource/.

## Supplementary material

For supplementary material accompanying this paper visit https://doi.org/10.1192/bjo.2020.75. click here to view supplementary material

## References

[1] Wang C, Horby PW, Hayden FG, Gao GF. A novel coronavirus outbreak of global health concern. Lancet 2020; 395: 470–3. 31986257 10.1016/S0140-6736(20)30185-9 PMC7135038

[2] World Health Organization. WHO COVID-19 Dashboard. WHO, 2020 (https://who.sprinklr.com/).

[3] Willan J, King AJ, Jeffery K, Bienz N. Challenges for NHS hospitals during covid-19 epidemic. BMJ 2020; 368: m1117. 32198166 10.1136/bmj.m1117

[4] Yao H, Chen JH, Xu YF. Patients with mental health disorders in the COVID-19 epidemic. Lancet Psychiatry 2020; 7: e21. 32199510 10.1016/S2215-0366(20)30090-0 PMC7269717

[5] Lamontagne-Godwin F, Burgess C, Clement S, Gasston-Hales M, Greene C, Manyande A, Interventions to increase access to or uptake of physical health screening in people with severe mental illness: a realist review. BMJ Open 2018; 8: e019412. 10.1136/bmjopen-2017-019412 PMC5829934 29440160

[6] Rodgers M, Dalton J, Harden M, Street A, Parker G, Eastwood A. Integrated care to address the physical health needs of people with severe mental illness: a rapid review. Heal Serv Deliv Res 2016; 4: 1–130. 27123505

[7] Björk Brämberg E, Torgerson J, Norman Kjellström A, Welin P, Rusner M. Access to primary and specialized somatic health care for persons with severe mental illness: a qualitative study of perceived barriers and facilitators in Swedish health care. BMC Fam Pract 2018; 19: 12. 29316894 10.1186/s12875-017-0687-0 PMC5759233

[8] Nease DE. Addressing the health care needs of patients with serious mental illness - it takes a system. J Prim Health Care 2014; 6: 6. 24624405

[9] Nankivell J, Platania-Phung C, Happell B, Scott D. Access to physical health care for people with serious mental illness: a nursing perspective and a human rights perspective-common ground. Issues Ment Health Nurs 2013; 34: 442–50. 23805929 10.3109/01612840.2012.754974

[10] Druss BG. Addressing the COVID-19 pandemic in populations with serious mental illness. JAMA Psychiatry 2020; 2019: 2019–20. 10.1001/jamapsychiatry.2020.0894 32242888

[11] Simonnet A, Chetboun M, Poissy J, Raverdy V, Noulette J, Duhamel A, High prevalence of obesity in severe acute respiratory syndrome coronavirus-2 (SARS-CoV-2) requiring invasive mechanical ventilation. Obesity 2020; 28: 1195–9. 32271993 10.1002/oby.22831 PMC7262326

[12] Sattar N, McInnes IB, McMurray JJV. Obesity a risk factor for severe COVID-19 infection: multiple potential mechanisms. Circulation 2020; 142: 4–6. 32320270 10.1161/CIRCULATIONAHA.120.047659

[13] Dietz W, Santos-Burgoa C. Obesity and its implications for COVID-19 mortality. Obesity 2020; 28: 1005–15. 32237206 10.1002/oby.22818

[14] Rubino S, Kelvin N, Bermejo-Martin JF, Kelvin DJ. As COVID-19 cases, deaths and fatality rates surge in Italy, underlying causes require investigation. J Infect Dev Ctries 2020; 14: 265–7. 32235086 10.3855/jidc.12734

[15] Holmes EA, O'Connor RC, Perry VH, Tracey I, Wessely S, Arseneault L, Multidisciplinary research priorities for the COVID-19 pandemic: a call for action for mental health science. Lancet Psychiatry 2020; 7: P547–60. 10.1016/S2215-0366(20)30168-1 PMC7159850 32304649

[16] Bycroft C, Freeman C, Petkova D, Band G, Elliott LT, Sharp K, The UK Biobank resource with deep phenotyping and genomic data. Nature 2018; 562: 203–9. 30305743 10.1038/s41586-018-0579-z PMC6786975

[17] UK BIObank. UK BIObank Makes Infection and Health Data. UK BIObank, 2020 (https://www.ukbiobank.ac.uk/2020/04/covid/).

[18] Davis KAS, Coleman JRI, Adams M, Allen N, Breen G, Cullen B, Mental health in UK Biobank – development, implementation and results from an online questionnaire completed by 157 366 participants: a reanalysis. BJPsych Open 2020; 6; e18. 32026800 10.1192/bjo.2019.100 PMC7176892

[19] Sudlow C, Gallacher J, Allen N, Beral V, Burton P, Danesh J, UK biobank: an open access resource for identifying the causes of a wide range of complex diseases of middle and old age. PLoS Med 2015; 12: e1001779. 25826379 10.1371/journal.pmed.1001779 PMC4380465

[20] Miller KL, Alfaro-Almagro F, Bangerter NK, Thomas DL, Yacoub E, Xu J, Multimodal population brain imaging in the UK Biobank prospective epidemiological study. Nat Neurosci 2016; 19: 1523–36. 27643430 10.1038/nn.4393 PMC5086094

[21] Pinzón-Espinosa J, *Enhancing Resilience in Psychosis Through Within and Between-Family Polygenic Risk Scoring, Gene x Gene Interactions and Gene-Environment (GxE) Prediction Models (REGENESIS)* UK BIObank, 2019 (https://www.ukbiobank.ac.uk/2019/11/enhancing-resilience-in-psychosis-through-within-and-between-family-polygenic-risk-scoring-gene-x-gene-interactions-and-gene-environment-gxe-prediction-models-regenesis/).

[22] UK BIObank. UKB: Data-Field 40100 - Records of COVID-19 Test Results. UK BIObank, 2020 (https://biobank.ndph.ox.ac.uk/showcase/field.cgi?id=40100).

[23] von Elm E, Altman DG, Egger M, Pocock SJ, Gøtzsche PC, Vandenbroucke JP. The Strengthening the Reporting of Observational Studies in Epidemiology (STROBE) statement: guidelines for reporting observational studies. J Clin Epidemiol 2008; 61: 344–9. 18313558 10.1016/j.jclinepi.2007.11.008

[24] World Health Organization. International Classification of Diseases (ICD-10). WHO, 2010 (https://icd.who.int/browse10/2010/en).

[25] R Core Team. R: A Language and Environment for Statistical Computing. R Core Team, 2019 (https://www.r-project.org/).

[26] Tyrrell J, Jones SE, Beaumont R, Astley CM, Lovell R, Yaghootkar H, Height, body mass index, and socioeconomic status: Mendelian randomisation study in UK Biobank. BMJ 2016; 352; i582. 10.1136/bmj.i582 PMC4783516 26956984

[27] Townsend P, Phillimore P, Beattie A. Health and Deprivation: Inequality and the North. Routledge, 1988.

[28] Malhi GS, Mann JJ. Depression. Lancet 2018; 392: 2299–312. 30396512 10.1016/S0140-6736(18)31948-2

[29] Giacco D, Palumbo C, Strappelli N, Catapano F, Priebe S. Social contacts and loneliness in people with psychotic and mood disorders. Compr Psychiatry 2016; 66: 59–66. 26995237 10.1016/j.comppsych.2015.12.008

[30] Jacob L, Haro JM, Koyanagi A. Relationship between living alone and common mental disorders in the 1993, 2000 and 2007 National Psychiatric Morbidity Surveys. PLoS One 2019; 14; e0215182. 31042720 10.1371/journal.pone.0215182 PMC6493731

[31] Matthews T, Danese A, Caspi A, Fisher HL, Goldman-Mellor S, Kepa A, Lonely young adults in modern Britain: Findings from an epidemiological cohort study. Psychol Med 2019; 49: 268–77. 29684289 10.1017/S0033291718000788 PMC6076992

[32] Poewe W, Seppi K, Tanner CM, Halliday GM, Brundin P, Volkmann J, Parkinson disease. Nat Rev Dis Prim 2017; 3: 1–21. 10.1038/nrdp.2017.13 28332488

[33] Silveira C, Guedes R, Maia D, Curral R, Coelho R. Neuropsychiatric symptoms of multiple sclerosis: state of the art. Psychiatry Investig 2019; 16: 877–88. 10.30773/pi.2019.0106 PMC6933139 31805761

[34] Scarioni M, Gami-Patel P, Timar Y, Seelaar H, van Swieten JC, Rozemuller AJM, Frontotemporal dementia: correlations between psychiatric symptoms and pathology. Ann Neurol 2020; 87: 950–61. 32281118 10.1002/ana.25739 PMC7318614

[35] Leyhe T, Reynolds CF, Melcher T, Linnemann C, Klöppel S, Blennow K, A common challenge in older adults: Classification, overlap, and therapy of depression and dementia. Alzheimer's Dement 2017; 13: 59–71. 27693188 10.1016/j.jalz.2016.08.007

[36] Keyes KM, Westreich D. UK Biobank, big data, and the consequences of non-representativeness. Lancet 2019; 393: 1297. 10.1016/S0140-6736(18)33067-8 PMC7825643 30938313

[37] Fry A, Littlejohns TJ, Sudlow C, Doherty N, Adamska L, Sprosen T, Comparison of sociodemographic and health-related characteristics of UK biobank participants with those of the general population. Am J Epidemiol 2017; 186: 1026–34. 28641372 10.1093/aje/kwx246 PMC5860371

[38] Abdellaoui A. Regional differences in reported Covid-19 cases show genetic correlations with higher socio-economic status and better health, potentially confounding studies on the genetics of disease susceptibility. medRxiv [Preprint] 2020 Available from: https://doi.org/2020.04.24.20075333.

