# Associations between psychiatric disorders, COVID-19 testing probability and COVID-19 testing results: findings from a population-based study – ERRATUM

**DOI:** 10.1192/bjo.2020.119

**Published:** 2020-11-13

**Authors:** Dennis van der Meer, Justo Pinzón-Espinosa, Bochao D. Lin, Joeri K. Tijdink, Christiaan H. Vinkers, Sinan Guloksuz, Jurjen J. Luykx

This article failed to state the coequal contribution to the article of its first two authors, Dennis van der Meer and Justo Pinzón-Espinosa. This note was deleted accidentally during the production process, for which the publisher apologises.
