# Supplementary material for: Associations between psychiatric disorders, COVID-19 testing probability and COVID-19 testing results: findings from a population-based study
Source: BJPsych Open. 2020 Jul 22;6(5):e87. doi: 10.1192/bjo.2020.75 (PMC7417998; doi:10.1192/bjo.2020.75)
Supplement: Supplementary file 1 [file S2056472420000757sup.zip › S2056472420000757sup001.docx]

**Supplemental information**

-

Associations between psychiatric disorders, COVID-19 testing probability and COVID-19 testing results

Findings from a population-based study

**Authors:** Dennis van der Meer, PhD^1,2,*^; Justo Pinzón-Espinosa, MD, MSc^3-6,*^; Bochao D. Lin, PhD^7,8^; Joeri K. Tijdink, MD, PhD^9,10^; Christiaan H. Vinkers^11,12^; Sinan Guloksuz, MD, PhD^13,14^; Jurjen J. Luykx, MD, PhD^15-17^

* These authors contributed equally.

1 NORMENT, Division of Mental Health and Addiction, Oslo University Hospital & Institute of Clinical Medicine, University of Oslo, Oslo, Norway

2 School of Mental Health and Neuroscience, Faculty of Health, Medicine and Life Sciences, Maastricht University, The Netherlands

3 Psychiatry Trainee, Barcelona Clínic Schizophrenia Unit, Department of Psychiatry and Psychology, Institute of Neuroscience, Hospital Clínic of Barcelona, Catalonia, Spain.

4 Predoctoral researcher, Department of Medicine, School of Medicine, University of Barcelona, Catalonia, Spain.

5 Predoctoral researcher, August Pi i Sunyer Biomedical Research Institute (IDIBAPS), Barcelona, Catalonia, Spain.

6 Ad-honorem Clinical Professor, Department of Clinical Psychiatry, School of Medicine, University of Panama, Panama City, Panama.

7 Post-doctoral researcher, Department of Psychiatry, Brain Center Rudolf Magnus, University Medical Center Utrecht, Utrecht University, Utrecht, the Netherlands

8 Post-doctoral researcher, Department of Translational Neuroscience, Brain Center Rudolf Magnus, University Medical Center Utrecht, Utrecht University, Utrecht, the Netherlands

9 Assistant professor, Department of Medical Humanities, Amsterdam University Medical Center, Amsterdam, the Netherlands

10 Assistant professor, Department of Philosophy, Vrije Universiteit, Amsterdam, the Netherlands

11 Associate professor, Department of Psychiatry, Amsterdam University Medical Center, Amsterdam, the Netherlands

12 Associate professor, Department of Anatomy and Neurosciences, Amsterdam University Medical Center, Amsterdam, the Netherlands

13 Assistant professor, Department of Psychiatry and Neuropsychology, School for Mental Health and Neuroscience, Maastricht University Medical Center, Maastricht, the Netherlands

14 Asssistant professor, Department of Psychiatry, Yale University School of Medicine, New Haven, Connecticut, USA

15 Assistant professor, Outpatient Second Opinion Clinic, GGNet Mental Health, Warnsveld, The Netherlands

16 Assistant professor, Department of Psychiatry, Brain Center Rudolf Magnus, University Medical Center Utrecht, Utrecht University, Utrecht, the Netherlands

17 Assistant professor, Department of Translational Neuroscience, Brain Center Rudolf Magnus, University Medical Center Utrecht, Utrecht University, Utrecht, the Netherlands

*Table S1. Conditions included in the analysis by ICD-10 diseases category. ICD-10 codes begin each disease category.*

Psychiatric (F00 – F99)

F00-09 Dementias and other types of brain disorders causing behavioral symptoms

F10-19 Substance use disorders

F20-29 Psychotic disorders

F32-39 Mood disorders – unipolar

F30-31 Mood disorders – bipolar

F40-41 Anxiety disorders

F42-48 Obsessive-compulsive disorder and stress-related disorders

F60-62, F68-69 Personality disorders

F70-79 Intellectual disability

F80-98 Developmental and other childhood-onset disorders

F50-F54; F63-F66; F99 Other psychiatric disorders

Neurological (G0x-G4x), i.e. specifically central nervous system disorders

[G00-G09](https://www.icd10data.com/ICD10CM/Codes/G00-G99/G00-G09)   Inflammatory diseases of the central nervous system

[G10-G14](https://www.icd10data.com/ICD10CM/Codes/G00-G99/G10-G14)   Systemic atrophies primarily affecting the central nervous system

[G20-G26](https://www.icd10data.com/ICD10CM/Codes/G00-G99/G20-G26)   Extrapyramidal and movement disorders

[G30-G32](https://www.icd10data.com/ICD10CM/Codes/G00-G99/G30-G32)   Other degenerative diseases of the nervous system

[G35-G37](https://www.icd10data.com/ICD10CM/Codes/G00-G99/G35-G37)   Demyelinating diseases of the central nervous system

[G40-G47](https://www.icd10data.com/ICD10CM/Codes/G00-G99/G40-G47)  Episodic and paroxysmal disorders

Circulatory (Ixx)

[I00-I02](https://www.icd10data.com/ICD10CM/Codes/I00-I99/I00-I02)   Acute rheumatic fever

[I05-I09](https://www.icd10data.com/ICD10CM/Codes/I00-I99/I05-I09)   Chronic rheumatic heart diseases

[I10-I16](https://www.icd10data.com/ICD10CM/Codes/I00-I99/I10-I16)   Hypertensive diseases

[I20-I25](https://www.icd10data.com/ICD10CM/Codes/I00-I99/I20-I25)   Ischemic heart diseases

[I26-I28](https://www.icd10data.com/ICD10CM/Codes/I00-I99/I26-I28)   Pulmonary heart disease and diseases of pulmonary circulation

[I30-I52](https://www.icd10data.com/ICD10CM/Codes/I00-I99/I30-I52)   Other forms of heart disease

[I60-I69](https://www.icd10data.com/ICD10CM/Codes/I00-I99/I60-I69)   Cerebrovascular diseases

[I70-I79](https://www.icd10data.com/ICD10CM/Codes/I00-I99/I70-I79)   Diseases of arteries, arterioles, and capillaries

Respiratory (Jxx)

J00-J06 Acute upper respiratory infections

J09-J18 Influenza and pneumonia

J20-J22 Other acute lower respiratory infections

J30-J39 Other diseases of upper respiratory tract

J40-J47 Chronic lower respiratory diseases

J60-J70 Lung diseases due to external agents

J80-J84 Other respiratory diseases principally affecting the interstitium

J85-J86 Suppurative and necrotic conditions of lower respiratory tract

J90-J94 Other diseases of pleura

J95-J99 Other diseases of the respiratory system

Metabolic (Exx)

[E00-E07](https://www.icd10data.com/ICD10CM/Codes/E00-E89/E00-E07)   Disorders of thyroid gland

[E08-E13](https://www.icd10data.com/ICD10CM/Codes/E00-E89/E08-E13)   Diabetes mellitus

[E15-E16](https://www.icd10data.com/ICD10CM/Codes/E00-E89/E15-E16)   Other disorders of glucose regulation and pancreatic internal secretion

[E40-E46](https://www.icd10data.com/ICD10CM/Codes/E00-E89/E40-E46)   Malnutrition

[E50-E64](https://www.icd10data.com/ICD10CM/Codes/E00-E89/E50-E64)   Other nutritional deficiencies

[E65-E68](https://www.icd10data.com/ICD10CM/Codes/E00-E89/E65-E68)   Overweight, obesity and other hyperalimentation

[E70-E88](https://www.icd10data.com/ICD10CM/Codes/E00-E89/E70-E88)   Metabolic disorders

***Overview of comorbidity***

In Table S2 we present an overview of the comorbidity rates between the ICD-10 diagnoses among individuals tested for COVID-19. Each cell indicates the proportion of individuals with the diagnosis specified in the first column who also have the diagnosis specified at the top of the subsequent columns.

*Table S2. Comorbidity between the ICD-10 diagnoses included in the analyses. Association between COVID-19 test outcome and ICD-10 diagnosis, covarying for respiratory, cardiovascular, and metabolic disease.*

|  | Psychiatric | Substance | Anxiety | Depression | Neurological | Respiratory | Cardiovascular | Metabolic |
| --- | --- | --- | --- | --- | --- | --- | --- | --- |
| Psychiatric disorder | 1 | 0.50 | 0.23 | 0.45 | 0.28 | 0.53 | 0.76 | 0.62 |
| - Substance use | 1 | 1 | 0.17 | 0.26 | 0.23 | 0.56 | 0.76 | 0.61 |
| - Anxiety | 1 | 0.38 | 1 | 0 .56 | 0.29 | 0.55 | 0.79 | 0.71 |
| - Depression | 1 | 0.29 | 0.29 | 1 | 0.35 | 0.55 | 0.74 | 0.63 |
| Neurological | 0.52 | 0.21 | 0.12 | 0.29 | 1 | 0.52 | 0.78 | 0.68 |
| Respiratory | 0.40 | 0.21 | 0.09 | 0.18 | 0.21 | 1 | 0.78 | 0.58 |
| Cardiovascular | 0.33 | 0.16 | 0.08 | 0.14 | 0.18 | 0.45 | 1 | 0.59 |
| Metabolic | 0.37 | 0.18 | 0.10 | 0.17 | 0.22 | 0.47 | 0.82 | 1 |

As a sensitivity analysis, we reran the primary analyses, predicting COVID-19 test outcome from ICD-10 diagnoses, with four additional covariates, namely diagnoses of respiratory, cardiovascular, and metabolic disease, and the Townsend deprivation index (as a measure of socio-economic status). The pattern of results was highly similar to the main results, as shown in Figure S1. Note, for the association of respiratory, cardiovascular, and metabolic disease diagnoses with test outcome we left out the covariate for that disease, i.e. we did not include the outcome as covariate.

*
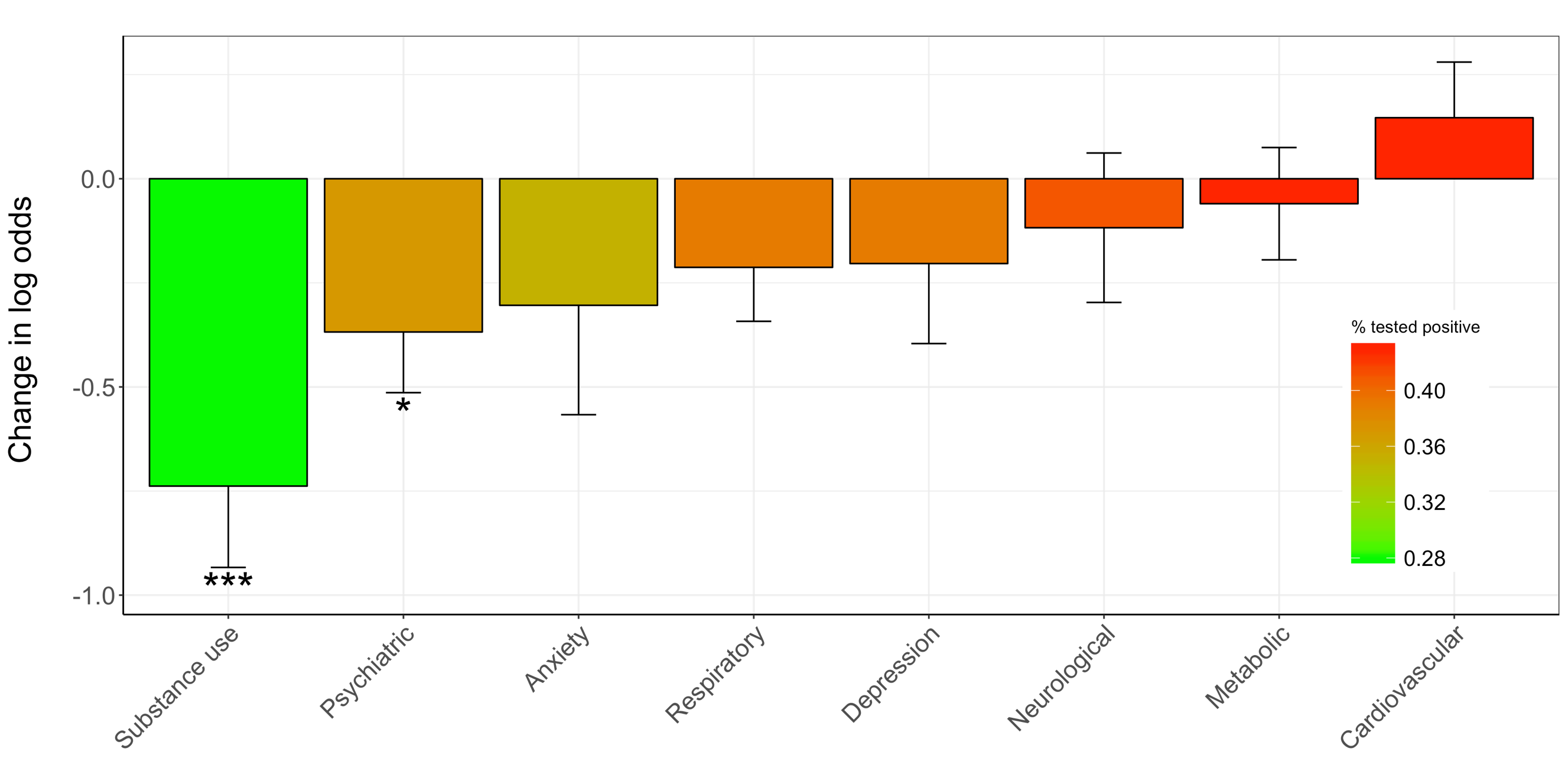
*

*Figure S1. Bar plot of odds ratios for testing positive, by ICD-10 diagnosis while additionally covarying for diagnoses of respiratory, cardiovascular, and metabolic disease diagnoses, and socio-economic status. Odds ratios are shown on the y-axis, on the x-axis are the diagnoses, colors indicate percent that tested positive. Note that ‘psychiatric’ category includes participants with substance, anxiety, and depressive disorders as those three are subcategories of the ‘psychiatric disorders’. Stars indicate significance: * p<* .*05, ** p<* .*005, ***p<.0005.*

*Associations between mental health items derived from the UKB mental health questionnaire and COVID-19 testing*

We additionally tested whether mental health in the general population, regardless of ICD-10 diagnoses, was associated with COVID-19 testing prevalence and outcome. For this, we categorized people based on whether they responded affirmative to the mental health questions listed in Table S3. We then analysed the data identical to the primary analyses of ICD-10 diagnostic categories using affirmative answers as representing being a case. Please see the main text for the main results.

*Table S3. Mental health questions used to categorize individuals. Within each category, we binarized individuals on whether they responded affirmative or not on any of the questions. For happiness we binarized based on a mean split. The numbers before the categories and questions indicate the UKB data repository field codes.*

*138: Depression*

20446 Ever had prolonged feelings of sadness or depression

20441 Ever had prolonged loss of interest in normal activities

*139: Mania*

20502 Ever had period extreme irritability

20501 Ever had period of mania / excitability

*140: Anxiety*

20421 Ever felt worried, tense, or anxious for most of a month or longer

20425 Ever worried more than most people would in similar situation

*141: Addiction*

20401 Ever addicted to any substance or behaviour

*144: Psychotic experiences*

20468 Ever believed in an un-real conspiracy against self

20474 Ever believed in un-real communications or signs

20463 Ever heard an un-real voice

20471 Ever seen an un-real vision

*146: Self-harm*

20479 Ever thought that life not worth living

20485 Ever contemplated self-harm

20480 Ever self-harmed

*147: Happiness*

20458 General happiness*

* Coded as follows:

1 Extremely happy

2 Very happy

3 Moderately happy

4 Moderately unhappy

5 Very unhappy

6 Extremely unhappy

We found no significant associations between test outcome and the mental health questions. Figure S2 and S3 summarize this; in Figure S2 we show the results with and without including individuals with a psychiatric disorder, and in Figure S3 we show the results after additionally covarying for respiratory, cardiovascular, and metabolic diagnoses, and socio-economic status.

**A**

*
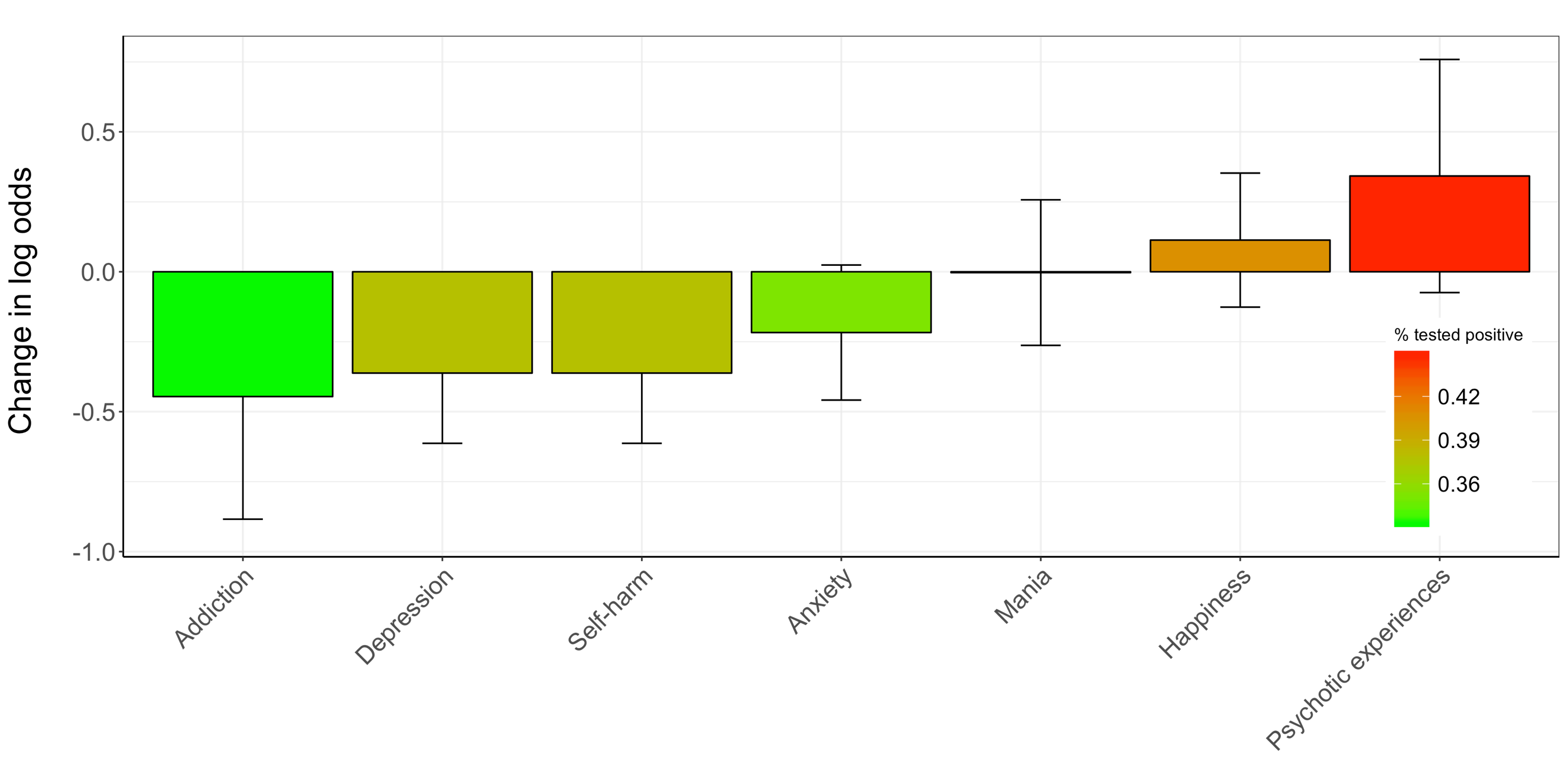
*

**B***
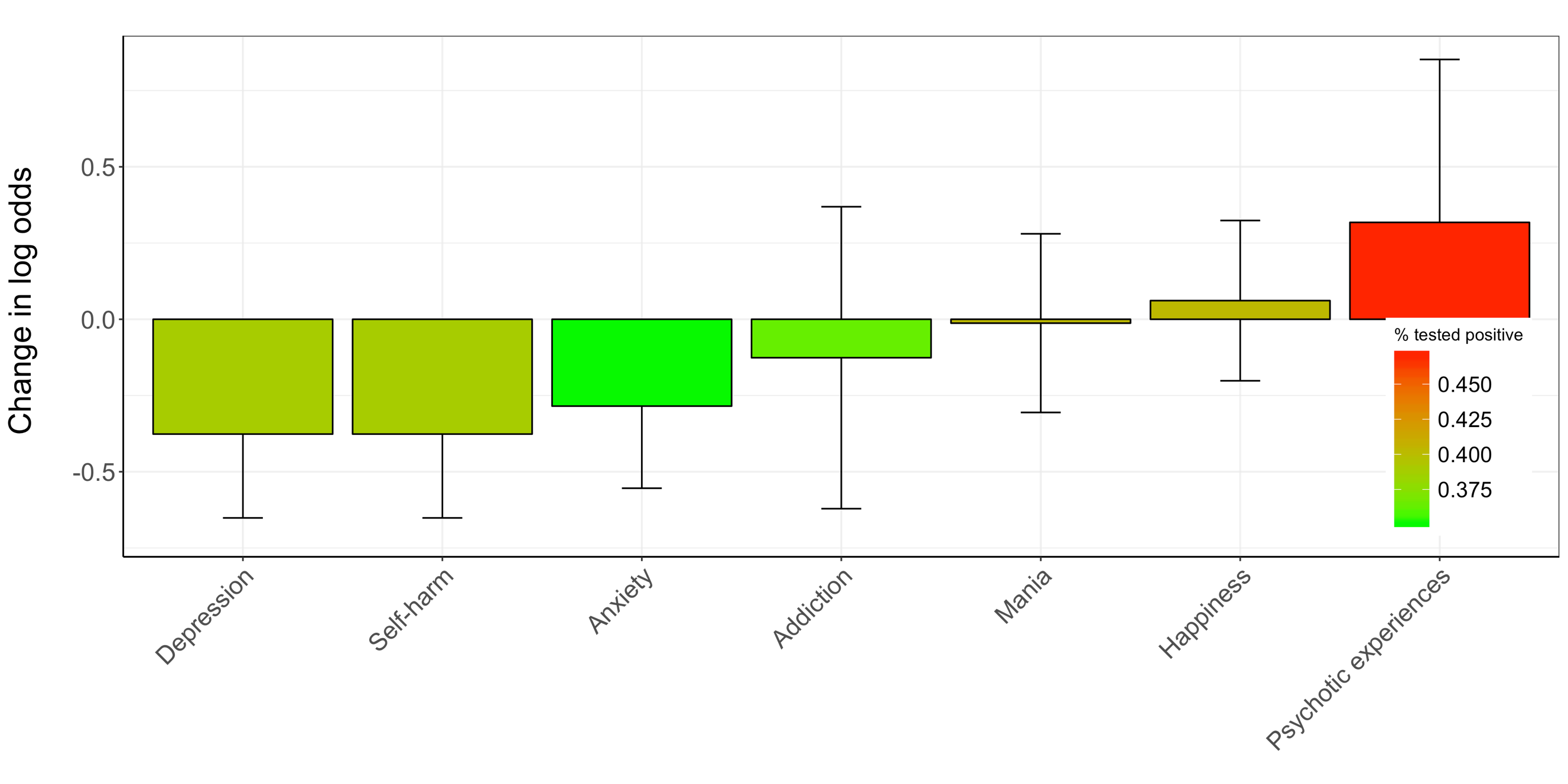
*

*Figure S2. Bar plots of odds ratios for testing positive, per mental health category based on affirmative responses to mental health questions in the mental health questionnaire of the UKB. Odds ratios are shown on the y-axis, on the x-axis are the categories, colors indicate percent that tested positive. A) is across the entire cohort with complete data, B) is after excluding those with a psychiatric disorder diagnosis.*

**A***
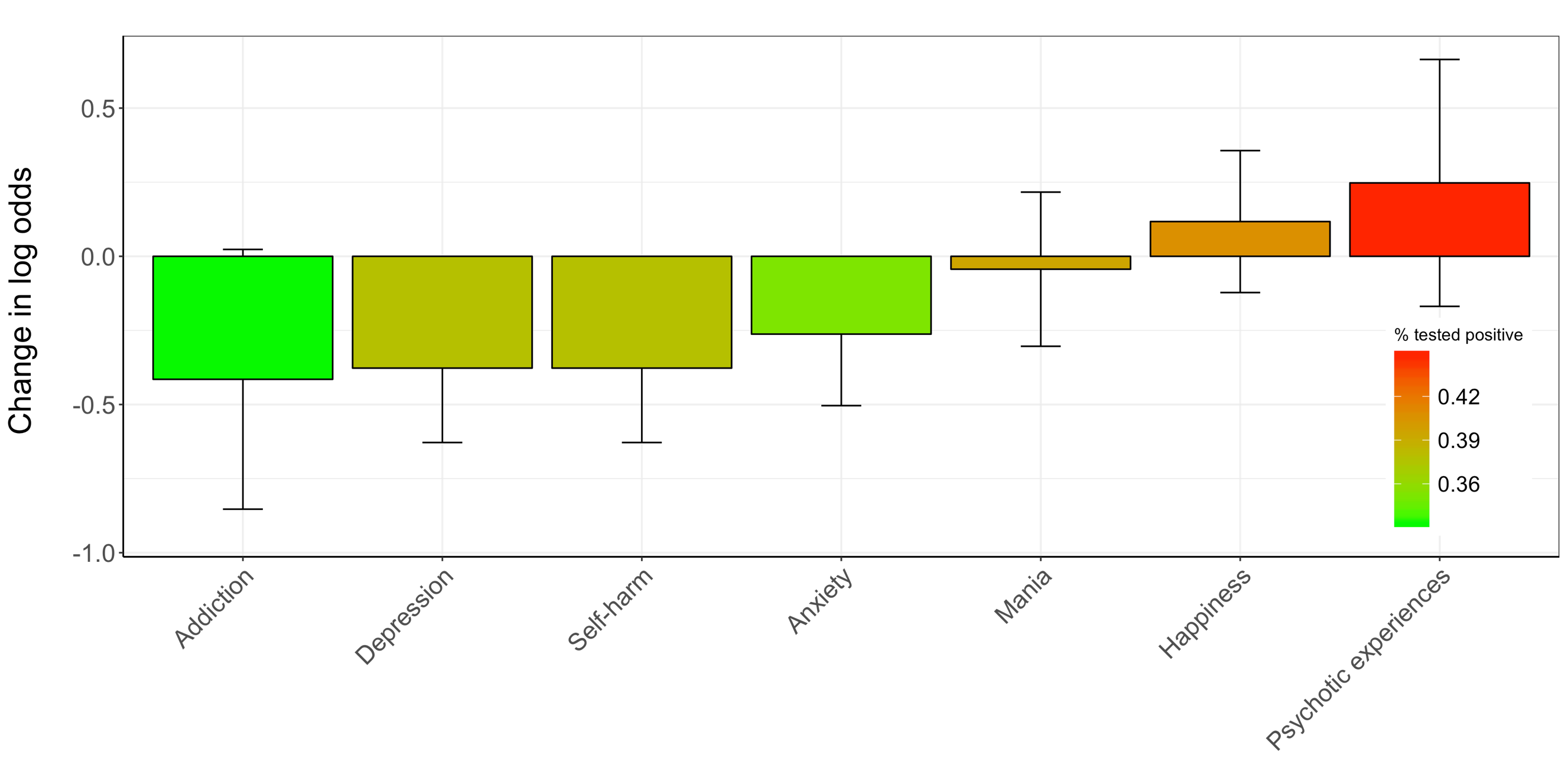
*

**B
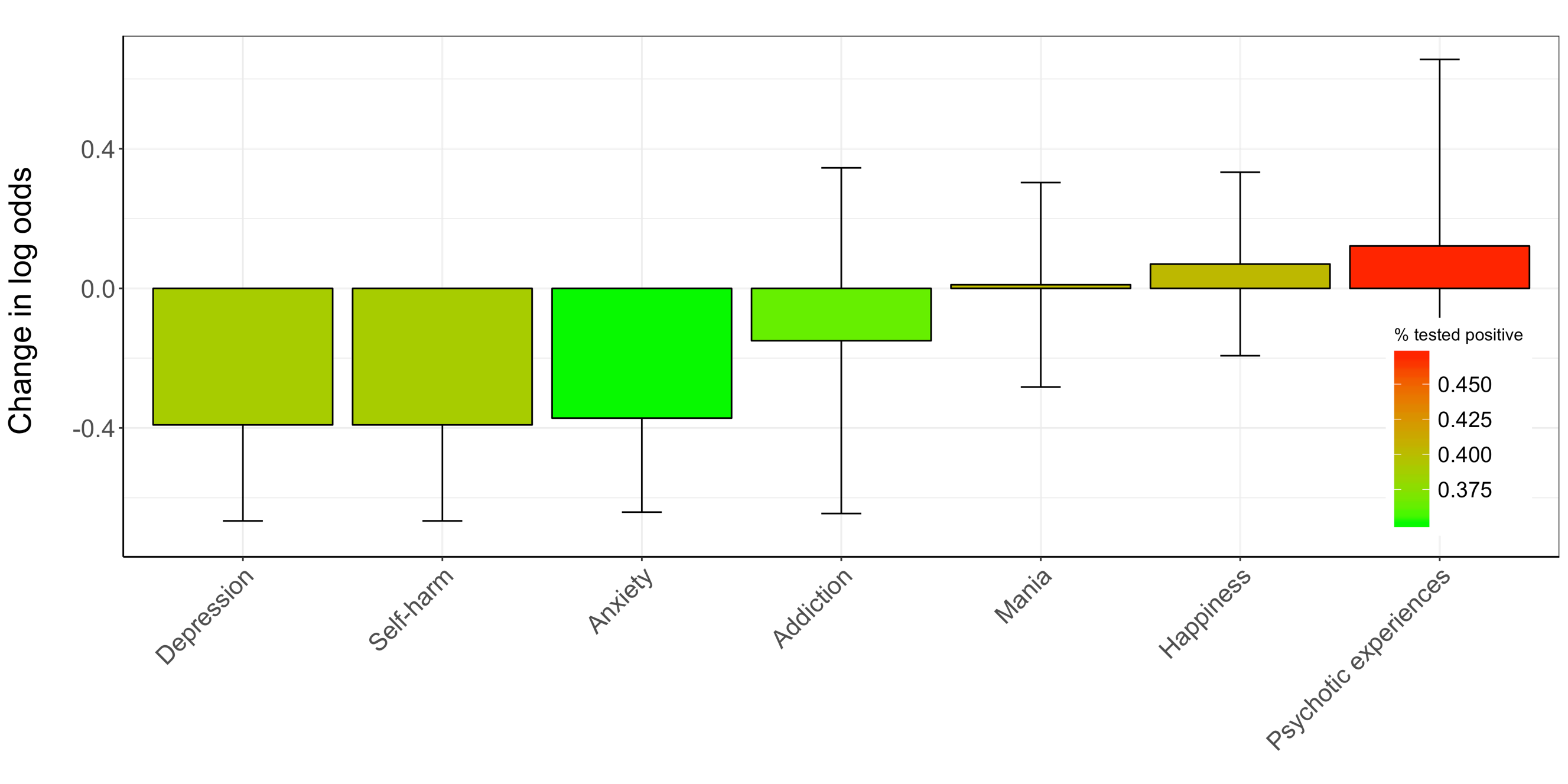
**

*Figure S3. Bar plots of odds ratios for testing positive, per mental health category based on affirmative responses to mental health questions in the mental health questionnaire of the UKB., additionally covarying for respiratory, cardiovascular, and metabolic diagnoses, and socio-economic status. Odds ratios are shown on the y-axis, on the x-axis are the categories, colors indicate percent that tested positive. A) is across the entire cohort with complete data, B) is after excluding those with a psychiatric disorder diagnosis*
